# Missed Opportunities for Vaccination in the Dominican Republic: Results of an Operational Investigation

**DOI:** 10.1155/2016/4721836

**Published:** 2016-10-12

**Authors:** Zacarías Garib, Aida Lucía Vargas, Silas P. Trumbo, Kathleen Anthony, Jose Luis Diaz-Ortega, Pamela Bravo-Alcántara, Irene Leal, M. Carolina Danovaro-Holliday, Martha Velandia-González

**Affiliations:** ^1^Expanded Programme of Immunization, Ministry of Public Health and Social Assistance, Santo Domingo, Dominican Republic; ^2^Vanderbilt University School of Medicine, Nashville, TN, USA; ^3^Instituto Nacional de Salud Pública, Cuernavaca, MOR, Mexico; ^4^Comprehensive Family Immunization Unit, Pan American Health Organization, 525 23rd Street NW, Washington, DC 20037, USA; ^5^Expanded Programme on Immunization, Department of Immunization, Vaccines and Biologicals (IVB), World Health Organization, Geneva, Switzerland

## Abstract

*Background*. Despite the success of the Dominican Republic's National Immunization Program, homogenous vaccine coverage has not been achieved. In October 2012, the country implemented a study on missed opportunities for vaccination (MOVs) in children aged <5 years.* Methods*. A cross-sectional study of 102 healthcare facilities was implemented in 30 high-risk municipalities. Overall, 1500 parents and guardians of children aged <5 years were interviewed. A MOV is defined as when a person who is eligible for vaccination and with no contraindications visits a health facility and does not receive a required vaccine. We evaluated the causes of MOVs and identified risk factors associated with MOVs in the Dominican Republic.* Results*. Of the 514 children with available and reliable vaccination histories, 293 (57.0%) were undervaccinated after contact with a health provider. Undervaccinated children had 836 opportunities to receive a needed vaccine. Of these, 358 (42.8%) qualified as MOVs, with at least one MOV observed in 225 children (43.7%). Factors associated with MOVs included urban geographic area (OR = 1.80; *p* = 0.02), age 1–4 years (OR = 3.63; *p* ≤ 0.0001), and the purpose of the health visit being a sick visit (OR = 1.65; *p* = 0.02).* Conclusions*. MOVs were associated primarily with health workers failing to request and review patients' immunization cards.

## 1. Introduction

The National Immunization Program (NIP) in the Dominican Republic provides routine vaccination free of charge in all health facilities operated by the Ministry of Public Health and Social Assistance (MSP). As of August 2012, the national schedule included antigens against 12 diseases: Hepatitis B, diphtheria, pertussis, tetanus,* Haemophilus influenzae* type b (Hib), measles, mumps, rubella, poliomyelitis, seasonal influenza, rotavirus, and severe forms of tuberculosis [1].

The NIP has reduced the incidence of all these diseases in the country. Nevertheless, coverage rates remain below 90%. In 2011, the country reported 84% third-dose coverage of the oral polio vaccine (OPV3), 80% coverage of Hepatitis B (Hep B3), and 88% first-dose coverage of measles-mumps-rubella vaccine (MMR1) [2]. Homogenous coverage has not been achieved, with 46 of 155 municipalities estimated to have third-dose coverage of diphtheria, tetanus, and pertussis vaccine (DTP3) within 50–79% and 10 estimated to have coverage <50% [3]. Causes of low coverage were unknown but suspected to relate to vaccine shortages at subnational levels and poor access and follow-up rather than to insufficient demand of immunization services [4].

To design a tailored plan to raise coverage levels, in October 2012, the NIP implemented a study on the causes of undervaccination among children who access health services in 30 high-risk municipalities, using the* Methodology for the Evaluation of Missed Opportunities for Vaccination (MOVs)* developed by the Pan American Health Organization (PAHO) [5, 6]. An opportunity for vaccination is considered missed when a person who is eligible for vaccination and has no contraindication to it visits a health facility and does not receive all required vaccines [7]. Among other features, the PAHO methodology contains operational definitions, guidelines on sample design, implementation, and analysis of results, and questionnaires to be adapted for study implementation [5]. The Dominican Republic was the first country to use PAHO's revised MOV methodology. In this study, we present the causes of MOVs identified following parental interviews and vaccination card assessment and propose strategies to reduce them. Data from a parallel survey of healthcare professionals will be presented elsewhere.

## 2. Material and Methods

### 2.1. Design

We performed a cross-sectional study in primary and secondary healthcare facilities from 1 to 15 October 2012.

### 2.2. Population

Population consisted of adults leaving a health facility accompanied by a child aged <5 years. If more than one adult was present, the adult with the closest relationship to the child was interviewed.

### 2.3. Sample Size and Selection of Participants

Because the MOV survey is an operational tool to identify missed opportunities, we focused on municipalities with low vaccination coverage levels and used purposeful quota sampling rather than probability sampling. Thus, the sample is only representative of children aged <5 years visiting selected health facilities on the day of the study [5].

The PAHO methodology provides guidelines for determining the number of interviews to be conducted and, of these, how many will be “effective interviews”—that is, those accompanied by the transcription of a vaccination card to verify the child's immunization status [5]. Using population estimates, we assumed a target population of 20,000 individuals, though the number of individuals visiting health facilities on a single day is unknown [8]. Based on a 2.5% margin of error, a 95% confidence level, the assumption that 50% of participants would answer questions accurately, and practical experience that approximately one-third of parent guardians visiting health facilities would possess their children's vaccination cards, we determined a sample size of 1500 individuals in order to obtain 500 effective interviews needed for determining MOVs. Since the results of the last census were not yet available, we estimated that 80% of the population lived in urban and 20% lived in rural areas [9]. We therefore established a quota of 1200 participants in urban areas and 300 in rural zones, so that these parameters could be applied to the participant quotas in urban and rural areas. Finally, we assumed that 80% of inhabitants would use primary care facilities and 20% would use secondary care facilities.

### 2.4. Healthcare Facilities

A two-stage sampling model was employed to select geographic areas and then healthcare facilities. Each of the country's 155 municipalities was assigned a “vulnerability” score based on public health indicators, vaccine-preventable surveillance data, and reported vaccination coverage rates. The 30 municipalities with the highest scores were selected in six of the country's eight regions. The total sample was then proportionally distributed according to population size of each of the 30 municipalities.

To determine the number of hospitals and primary health facilities to include in the study and based on national experience, we estimated that 20 children aged <5 years visit hospitals each day and that 15–20 children visit primary care facilities each day. From a catalog of health facilities by municipality, 102 of 1503 (6.8%) healthcare facilities were randomly selected to participate in the MOV survey.

### 2.5. Implementation

The NIP and PAHO contracted a company with experience in implementing health surveys to perform exit interviews in health facilities. NIP and PAHO officials participated in all stages of the study.

PAHO's questionnaire was adapted for implementation in the Dominican Republic, taking into account local differences in vocabulary and the country's immunization schedule. Due to their recent introduction in the national schedule, rotavirus and influenza vaccines were excluded from evaluation [5]. The surveying tools assessed the administration of BCG (one dose), Hepatitis B (HB), OPV, DTP-Hib-HB, or whole-cell pentavalent (three doses at ages 2, 4, and 6 months), MMR (one dose recommended at 12 months), and DTP and OPV boosters (recommended at 18 months and 4 years). Given that shortages of pentavalent vaccine have occurred and that the NIP has used DTP and the Hib-HB (Comvax) as substitutes for pentavalent vaccines, we adjusted the surveying tools to account for this possibility.

NIP and PAHO staff trained interviewers on the study's objectives, topics in immunization, and content of the questionnaire. The questionnaire was then piloted in two hospitals in Santo Domingo. Prior to implementation, NIP leadership requested the cooperation of the directors of the health facilities via an official letter. To minimize biases, the exact dates of the interviews were announced only a few days before implementation.

The questionnaire contained a total of 48 questions pertaining to demographics (17), parental attitudes favoring immunization (4), the impact of communication strategies (6), immunization practices and MOVs (11), and service quality (10). To ascertain MOVs, participants were asked if their child had been vaccinated at the health facility. In cases in which a participant had more than one child aged <5 years, only information on the youngest child was obtained. Those participants answering “yes” about vaccination in the health facility were asked about the quality of service. Those answering “no” were asked why their child had not been vaccinated. Interviewers recorded the date and dose of all vaccines in the child's immunization history. Acceptable sources for this information included vaccination cards and other forms of documentation issued by health facilities. Responses based on parental recall were not accepted.

In primary health facilities, interviewers approached potential participants exiting the clinics. In secondary health facilities (hospitals), interviewers situated themselves near the main entrance, emergency room, pediatrics departments, and vaccination center. Interviewers attained informed verbal consent from participants, encouraged them to ask questions if they did not understand any part of the survey, and carefully transcribed the child's vaccination card. Interviews were conducted in Spanish and lasted 5–15 minutes.

### 2.6. Data Analysis

All data were entered twice into the Statistical Package for the Social Sciences Software (SPSS) and cross-checked for recording errors.

We established the following definitions:

 Eligible children: undervaccinated or unvaccinated children with no contraindications for receiving one or more doses of a required vaccine
 Vaccinated: children up-to-date with all required vaccines for their ages
 Undervaccinated: children missing one or more doses of a recommended vaccine
 Unvaccinated: children missing all doses of all recommended vaccines

MOVs in children lacking multiple vaccines were evaluated by vaccine type. We used an algorithm to assess the vaccines the child should have already received and those the child lacked upon visiting the health center. The algorithm took into account the child's age and each vaccine he or she should have received according to the national immunization schedule. Missing vaccines not administered during the visit were classified as MOVs and grouped into categories of reasons related to the caregiver, health staff, or health system. Of note, MOVs were assessed by vaccine, such that a single child may have had multiple MOVs (e.g., missed DTP and missed OPV). All statistical analyses were performed using SPSS and data are presented with 95% confidence intervals (CI);* p* values <0.05 were considered significant.

### 2.7. Ethical Considerations

The Ministry of Health approved the study and exempted it from formal review by the country's ethical committee, since the study was considered operational research to guide the work of the NIP. Potential participants were advised that the survey was anonymous and that they would receive no compensation, and they provided verbal informed consent.

## 3. Results

### 3.1. Description of the Sample

We identified 1564 adults or parent guardians with at least one child potentially eligible for the study. Of these, 1500 (95.9%) agreed to participate. Overall, 1221 (81.4%) interviews were conducted in urban areas and 1407 (93.8%) were conducted in health facilities under the MSP; 939 (62.6%) interviews occurred in hospitals. Of the 561 interviews in primary health facilities, 314 (20.9%) were in urban areas and 247 (16.5%) were in rural areas.

Of the 1500 children in the study, 756 (50.4%) were male and 640 (42.7%) were aged <1 year.  Table 1 presents the sociodemographic characteristics of the study participants. The most common reasons for visiting the healthcare facility include the child's illness (754, 50.3%), vaccination (363, 24.2%), or the caregiver's health consultation (243, 16.2%).

### 3.2. Vaccination Status and Missed Opportunities for Vaccination

Of 527 children with vaccination cards, 13 were excluded due to data inconsistencies and resulting concerns about data reliability. Of the remaining 514, 221 were vaccinated (up-to-date) and 293 were undervaccinated following contact with the health center. There was a general downward trend between age and the proportion of children with up-to-date immunization schedules; children aged <1 year were 2.29 times (95% CI = 1.58–3.33) more likely to be up to date compared to children aged 1–4 years. The proportion vaccinated ranged from 55.0% (at least one OPV booster) to 95.1% (BCG) (Table 2).

In all, undervaccinated children had 836 opportunities to receive needed vaccines. Of these, 358 (42.8%) qualified as MOVs, with five reported in BCG, 17 in HepB3, 47 in MMR, 88 in OPV, and 145 in a DTP-containing vaccine (Table 3). The remaining 478 opportunities were used to administer at least one dose of needed vaccine. Rates of MOV ranged from 11.4% in BCG to 52.7% for DTP-containing vaccines. One or more MOVs occurred in 225 children, with 103 (45.8%) of these receiving some but not all of the needed vaccines on the day of the survey.

MOVs were grouped into three categories: 49 (41.8%) related to healthcare personnel, 48 (41.0%) related to caregivers, and 20 (17.1%) related to the organization of the health system. Among reasons related to health workers, 37 (75.5%) participants said the doctor or nurse had told them that their child was up to date and did not need any additional vaccine(s) and 12 (24.5%) said the healthcare professional said the child could not be vaccinated due to illness. False contraindications included cold/cough, mild fever, malnutrition and/or anemia, and stomach pain. Reasons associated with the caregiver were related to the belief that the child did not need vaccines (22, 45.8%) or that they had already completed the immunization schedule (12, 25.0%) or other or not specified reasons (14, 29.1%). Among the 20 persons with reasons related to the health system, 17 attributed the lack of vaccination to vaccine stock-outs.

Factors associated with MOVs included urban geographic area (OR = 1.80; *p* = 0.02), age 1–4 years (OR = 3.63; *p* < 0.0001), and the purpose of health visit being a sick visit (OR = 1.65; *p* = 0.02) (Table 4).

### 3.3. Information on Immunization

When participants were asked how they obtained information on vaccination, 1077 (71.8%) said they obtained it at health facilities and 129 (8.7%) said they obtained it on the vaccination card; 188 (12.5%) said they did not seek out information on vaccination. However, 656 (43.7%) participants said that they lacked necessary information on immunization. Regarding the importance of vaccines, 1415 (94.3%) said their children, if not immunized, could contract vaccine-preventable diseases, while 85 (5.6%) said they did not believe or know if this was the case.

### 3.4. Immunization Practices and Service Quality

Of the 1467 (97.8%) participants who claimed to have a vaccination card for their child, 527 (35.1%) were in possession of the card at the time of interview. “Because I did not bring my child to be vaccinated” (653, 67.1%) and “because I left the card at home” (202, 20.8%) were the most common reasons given for not having the card. Cards were less available for older children (<1 year: 50% did not have card; 1–4 years: 76%; OR = 3.15 and *p* < 0.0001). A third of the participants (496, 33.1%) reported being asked for their child's card at the health facility.

On the day of the survey, 334 caregivers (22.3%) indicated their children had been vaccinated. Of these, 317 (94.9%) were satisfied with the service, citing kind treatment of health workers (61.8%) and immediate attention (34.4%). Two-hundred and seven (62.0%) respondents indicated they received information on adverse events, 305 (91.3%) said their child's next appointment was recorded on the vaccination card, and 161 (48.2%) said they were informed about the necessity of next doses. Approximately 5% (*n* = 94) of participants indicated they had been unable to vaccinate their children on at least one occasion. Almost all respondents indicated that health facilities provided vaccines free of charge (1479, 99%).

## 4. Discussion

In this study, causes of undervaccination and MOVs were related to health personnel (41.8%) and caregivers (41.0%). An important finding is that health workers do not request the vaccination card, though this practice has been recommended. Roughly one-third of the study participants had the vaccination card with them on the day of the interview. A similar proportion indicated that health workers asked for the card during their visit. These findings differ from those of a previous study on undervaccination conducted in the Dominican Republic in 2001-2002. In that study, problems related to the health system, such as vaccine storages, closed health facilities, and long wait times, were identified as the major barriers to immunization [4].

Had the guardians of the remaining two-thirds of children brought their cards and had those cards been properly reviewed and had the children been vaccinated accordingly, missed opportunities would have been reduced. Among children for whom the cause of MOVs could be determined, 60.6% were not vaccinated because the caregiver or health worker mistakenly believed the child to be fully vaccinated for his/her age. Furthermore, among the 303 children who received one or more vaccines on the day of the interview, 103 (34.0%) still presented at least one MOV, suggesting that, in at least some cases, the health worker improperly assessed the vaccination card and the vaccines still needed.

Our findings indicate that children run a greater risk of becoming undervaccinated as they age and must comply with multidose vaccine series and as they fall behind on vaccines that should be administered in the first year of life. In this study, significant proportions of children aged 1–<5 years had MOVs for vaccine doses that they should have received when they were aged <1 year, possibly skewing MOV results in older children. Even so, compared to MOV incidence in single-dose vaccines (e.g., BCG, 11.4%), the greater frequency of MOVs in multidose vaccines, such as DTP-containing vaccines (52.7%), suggests that parents and healthcare professionals may perceive immunization as less important for older children. Some studies have shown a greater risk for undervaccination and MOVs in older children, presumably due to the decreased perception of the importance of vaccination in this age group and complacency [10–12]. However, this might be context-specific, as other studies have shown greater risk in younger children [13–16]. The actual reasons for the differential rate of MOVs with age may need to be explored in further detail.

In a meta-analysis of 41 studies on MOV in low- and middle-income countries, Sridir et al. found a median prevalence of 32.2% in children, compared to the 43.7% in our study [17]. There are several explanations for this difference, most significantly that many studies in the meta-analysis only measured MOVs in children aged <2 years [17]. Regardless, a greater incidence of MOVs in the Latin American and Caribbean (LAC) Region has been reported. In a review of 14 studies of MOV in LAC prior to 2001, the median prevalence of MOVs was 50% (14–77%) [18]. Our results, though limited to vulnerable municipalities in one country, suggest that MOVs remain a major problem, at least in the Dominican Republic.

In the literature from Latin America, factors associated with MOVs have been mostly attributable to health workers and health systems [19–25], vaccine shortages [18–26], and the application of injectable immunogens that require multiple doses, such as Hep B and pentavalent vaccine [27]. Cultural barriers, the fear of adverse events attributable to vaccination, and difficulties in accessing health services have also been associated with MOVs and untimely vaccination [28, 29]. The magnitude and causes of MOV may vary by country and in different areas in a country. Thus, conducting similar MOV studies may help national authorities assess their situations and tailor solutions to their contexts.

Diverse strategies have been proposed to promote timely vaccination and decrease MOVs [30–32]. The results of this study resulted in health authorities in the Dominican Republic devising communication strategies aimed at promoting vaccination, particularly in urban areas, and activities to better inform caregivers and health workers about the need to continue monitoring and promoting vaccination among children aged 1–4 years. Most importantly, health authorities should advocate that caregivers keep and carry the vaccination card and that healthcare workers review it, with special communication and education activities designed to this effect [33]. Health workers must be properly trained and supervised to properly assess the child's card, paying particular attention to the completeness of multidose vaccine series, including the recommended booster doses. Training efforts could begin in municipalities with the lowest vaccination coverage and include follow-up activities to ensure compliance, as this is likely to result in measurable improvements. We recommended that this study, using the same methodology to evaluate MOVs, be repeated in 2017 to evaluate the effectiveness of these interventions.

Several limitations to this study should be recognized. The results are representative of the municipalities evaluated but not of the country overall because municipalities were selected based on vulnerability indicators rather than randomly. The study had a high rate of participation; however, recall and reporting biases may have affected participant responses to questions on service quality and attitudes about vaccination. Owing to the complexity of the vaccination schedules, we acknowledge that errors in the ascertainment of immunization status for age and MOVs may have occurred. Finally, as is the nature of MOV studies, this survey was implemented at health facilities and could not evaluate causes of undervaccination related to access to healthcare. In spite of these limitations, this study identified that children in the Dominican Republic are not receiving all needed vaccines despite contact with health facilities.

In 2013, the results of this study were shared with the health staff responsible for immunization in all provinces in the Dominican Republic, who then developed tailored plans aimed at reducing missed opportunities and barriers to immunization in their municipalities. Also, this study validated PAHO's protocol and surveying instruments as valuable tools for countries in the Americas wishing to conduct real-time evaluations of their immunization programs. In March 2013, PAHO made these tools available to all countries in the LAC Region [5]. By the end of 2015, Colombia, Panama, and Peru had conducted MOV studies using the PAHO protocol.

## 5. Conclusion

In the Dominican Republic, users recognize the importance of vaccines and are satisfied with immunization services. Based on this study, MOVs are associated primarily with parents who do not bring the child's vaccination card to health facilities and with health workers who fail to request the patient's immunization history despite his or her contact with the health system. These errors can be corrected with simple, cost-effective strategies that institutionalize the proper use of the vaccination card.

## Figures and Tables

**Table 1:** Sociodemographic characteristics of participants: Dominican Republic, October 2012.

Characteristic	Participants (*n* = 1500) number (%)
Parents or guardians	
*Sex *	
Female	1431 (95.4)
*Geographic area *	
Urban	1221 (81.4)
Rural	279 (18.6)
*Age*	
15–24	688 (45.9)
25–34	576 (38.4)
35–49	185 (12.3)
>50	51 (3.4)
*Relation to child*	
Mother/father	1341 (89.4)
Grandparent	92 (6.1)
Aunt/uncle	40 (2.7)
Other	27 (1.8)
*Education*	
Less than primary	413 (27.5)
Primary only	111 (7.4)
Secondary incomplete	381 (25.4)
Secondary or more	595 (39.7)
*Marital Status*	
Single	211 (14.1)
Married	231 (15.4)
Civil union	944 (62.9)
Separated/divorced	105 (7.0)
Widowed	9 (0.6)
*Occupation*	
Housewife	1050 (70.0)
Employee/laborer	257 (17.1)
Student	75 (5.0)
Other	118 (7.9)
*Reason for visiting health center*	
Child's sickness	754 (50.3)
Vaccination	363 (24.2)
Caregiver's consultation	243 (16.2)
Child's wellness visit	57 (3.8)
Other	83 (5.5)

Children in study	
*Sex *	
Female	744 (49.6)
Male	756 (50.4)
*Age *	
<2 months	151 (10.1)
2-3 months	117 (7.8)
4-5 months	117 (7.8)
6–11 months	255 (17.0)
1-2 years	370 (24.7)
3-4 years	490 (32.7)

**(a):** 

By age	Vaccination status				
	Total	Vaccinated (full vaccination for age)		Undervaccinated (incomplete vaccination for age)	
	*n*	*n*	%	*n*	%
<2 months	76	50	65.8	26	34.2
2-3 months	77	47	61	30	39
4-5 months	69	32	46.4	37	53.6
6–11 months	89	29	32.6	60	67.4
*Subtotal <1 year*	*311*	*158*	*50.8*	*153*	*49.2*
1 year	122	36	29.5	86	70.5
2 years	29	7	24.1	22	75.9
3 years	14	8	57.1	6	42.9
4 years	38	12	31.6	26	68.4
*Subtotal 1–4 years*	*203*	*63*	*31*	*140*	*69*

*Total*	*514*	*221*	*43*	*293*	*57*

**(b):** 

By vaccine	Vaccination status				
	Total	Vaccinated		Undervaccinated or not vaccinated	
	*n*	*n*	***%***	*n*	***%***
BCG	514	489	95.1	25^*∗*^	4.9
Hep-B birth dose	514	407	79.2	107^*∗*^	20.8
Min 1 dose OPV^1^	438	414	94.5	24^*∗*^	5.5
Min 1 dose of DPT^1,2^	438	414	94.5	24^*∗*^	5.5
Min 1 pentavalent dose^1^	438	397	90.6	41^*∗*^	9.4
MMR/MR^3^	203	157	77.3	46^*∗*^	22.7
Min 1 DPT booster^3^	131	75	57.3	56^*∗∗*^	42.7
Min 1 OPV booster^3^	131	72	55	59^*∗∗*^	45

^*∗*^Not vaccinated.

^*∗∗*^Undervaccinated.

^1^Excluding infants < 2 months.

^2^Including 1 dose of DTP-containing vaccine.

^3^Excluding infants < 1 year.

**Table 3:** Missed opportunities for vaccination by age and vaccine type: Dominican Republic, October 2012^*∗*^.

Characteristic	Total opportunities	Missed opportunities for vaccination	
	*N*	*n*	*%*
By age			
<2 months	86	22	25.6
2-3 months	121	25	20.7
4-5 months	107	36	33.6
6–11 months	144	61	42.4
*Subtotal <1 year*	*458*	*144*	*31.4*
1-2 years	232	136	58.6
2-3 years	48	37	77.1
3-4 years	15	8	53.3
4–<5 years	83	33	39.8

*Subtotal 1–4 years*	*378*	*214*	*56.6*

*Total*	*836*	*358*	*42.8*

By vaccine			
BCG	44	5	11.4
Hep-B RN	42	17	40.5
OPV	230	88	38.3
Pentavalent^*∗*^	275	145	52.7
MMR	95	47	49.5
DPT booster	69	21	30.4
OPV booster	81	35	43.2

*Total*	*836*	*358*	*42.8*

^*∗*^Pentavalent: it includes its equivalent (DPT + Comvax).

**Table 4:** Risk factors for missed opportunities for vaccination among children with recorded vaccination data: Dominican Republic, October 2012.

Risk factors	Total	MOV (*n* = 225) number (%)		Odds ratio (95% CI interval)	*p* value
*Age*					
<1 year	311	98	32%	1	**<0.0001**
1–4 years	203	127	63%	**3.63 (2.50–5.27)**	
*Geographic area*					
Rural	81	26	32%	1	**0.02**
Urban	433	199	46%	**1.80 (1.09–2.98)**	
*Type of establishment*					
Hospital	320	134	42%	1	0.27
Clinics	194	91	47%	1.23 (0.86–1.76)	
*Age of caregiver*					
<25 years	238	95	40%	1	0.1
>25 years	276	130	47%	1.34 (0.94–1.9)	
*Sex of child*					
Male	254	108	43%	1	0.57
Female	260	117	45%	1.11 (0.78–1.57)	
*Education*					
Secondary or more	348	156	45%	1	0.44
Primary or less	151	62	41%	0.86 (0.58–1.26)	
*Caregiver occupation* ^*∗*^					
Does not work	466	204	44%	1	0.91
Works	38	17	45%	1.04 (0.54–2.02)	
“*During this visit to the health center, did they vaccinate your child?*”					
Yes	313	108	35%	1	**<0.0001**
No	201	117	58%	**2.64 (1.84–3.81)**	
“*Did a health worker ask you for a vaccination card today?*”					
Yes	371	140	38%	**1**	**<0.0001**
No^*∗∗*^	143	85	59%	**2.42 (1.63–3.59)**	
*“For what reason did you come to this healthcare establishment?”*					
Vaccination	330	133	40%	1	—
Medical consult (“child is sick”)	129	68	53%	**1.65 (1.10–2.49)**	**0.02**
Well-child visits	24	13	54%	1.75 (0.76–4.02)	0.19
Other	31	11	35%	0.81 (0.38–1.76)	0.6

^*∗*^Caregiver occupation: only mothers measured.

^*∗∗*^Including “No, but they asked me about my child's vaccines” (*n* = 5).
